# Palmitoylethanolamide, an endogenous fatty acid amide, and its pleiotropic health benefits: A narrative review

**DOI:** 10.7555/JBR.38.20240053

**Published:** 2024-10-22

**Authors:** Debasis Basu

**Affiliations:** Healious Global METTA Clinic, Kolkata, West Bengal 700029, India

**Keywords:** nutrition, palmitoylethanolamide, health, cardiovascular disease, metabolic disease, diabetes

## Abstract

The global nutritional transition has led to the increased frequency and severity of chronic degenerative diseases worldwide, primarily driven by chronic inflammatory stress. At mealtimes, various pharmaceutical products aim to prevent such inflammatory stress, but they usually cause various systemic side effects. Therefore, the supplementation of natural and safe ingredients is a promising strategy to reduce the risk and severity of inflammatory stress-related diseases. Palmitoylethanolamide (PEA), an endocannabinoid-like mediator, has been extensively studied for its diverse actions, including anti-inflammatory, antimicrobial, immunostimulatory, neuroprotective, and pain-reducing effects, with high tolerability and safety in both animals and humans. Because of its multiple molecular targets and mechanisms of action, PEA has demonstrated therapeutic benefits in various diseases, including neurological, psychiatric, ophthalmic, metabolic, oncological, renal, hepatic, immunological, rheumatological, and gastrointestinal conditions. The current review highlights the roles and functions of PEA in various physiological and pathological conditions, further supporting its use as an important dietary agent.

## Introduction

Dietary nutrition plays a pivotal role in providing energy and essential building materials for maintaining optimal body health. Recent research has highlighted the molecular and mechanistic roles of nutrition in various biological activities and body functions^[1]^. However, the shift in human dietary patterns from natural and nutritional diets to processed and nutritionally deficient diets has created a "nutritional pandemic", which is considered one of the major risk factors for the development of various chronic diseases^[2–3]^. Concurrently, the change in lifestyle, including increased automation and sedentary behaviors, as well as environmental factors, such as rising levels of pollutants, further amplifies the negative effects of this nutritional transition, increasing conditions like obesity, dyslipidemia, metabolic syndrome (MetSyn), cardiovascular diseases, bone and joint complications, neurological disorders, oncologic diseases, and others^[4–5]^. A recent study has also underscored the potential role of nutrition at the gene expression level (termed nutrigenomics) and the subsequent role of the genome in the nutritional requirements (termed nutrigenetics), potentially linking the nutritional pandemic to hereditary disease conditions^[6]^.

The rise in chronic diseases subsequently increases the consumption of pharmaceutical medicines either as prophylactic therapy, treatment options, or for maintaining body health^[7–8]^. Multiple medicine use (polypharmacy) is also associated with other risks, including drug resistance, tolerance, side effects, and increased financial burdens^[8–9]^. These challenges underscore the need for an alternative therapeutic approach that minimizes adverse effects and costs while maximizing efficacy. Natural food derivatives and constituents offer promising avenues for managing chronic diseases and enhancing overall health^[10]^. Among these, palmitoylethanolamide (PEA), an endocannabinoid-like mediator, has garnered attention for its multifaceted therapeutic properties. The endocannabinoid system, comprising receptors (mainly cannabinoid receptors 1 and 2 [CB_1_ and CB_2_]), endogenous ligands (endocannabinoids), and degrading enzymes, plays a pivotal role in various physiological processes^[10]^.

## PEA: An overview

### Structure and pharmacokinetics

**Figure 1:**
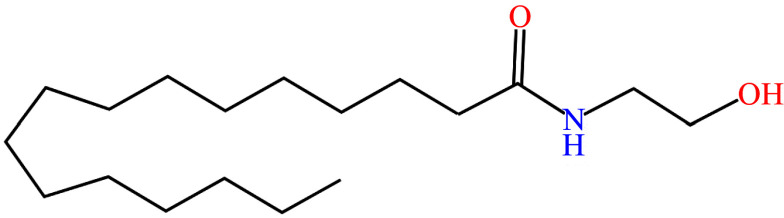
Structure of palmitoylethanolamide.

**Figure 2:**
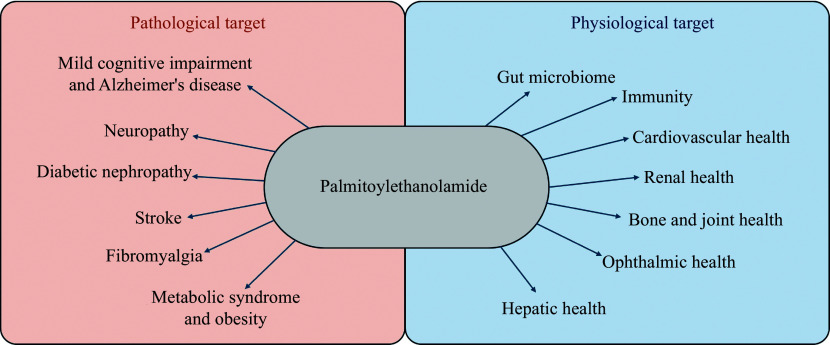
Physiological and pathological targets of palmitoylethanolamide.

The bioactive lipid mediator PEA (chemical structure shown in ***Fig. 1***) is a member of the N-acyl-ethanolamine (NAE) fatty acid amide family and resembles endocannabinoids. PEA is synthesized within the lipid bilayer ''on demand'' and is present in almost all tissues of the body^[10]^. PEA is typically elevated in disease states as a pro-homeostatic defense response against cellular damage^[11]^. Various molecular studies have verified that PEA has numerous actions, including strong anti-inflammatory and pain-relieving effects, anticonvulsant effects, antimicrobial effects, antiepileptic effects, immunomodulatory effects, and neuroprotective effects^[10]^. Because of its multiple mechanisms of action, PEA may provide therapeutic benefits in many diseases across various body systems, including neurology, psychiatry, ophthalmology, metabolic disorders, oncology, renal, hepatic, immune, joint, and gut (***Fig. 2***).

PEA is naturally present in various food sources, including soy lecithin, roasted coffee, black-eyed peas, apples, lentils, and potatoes, albeit in small quantities. It is also found in human milk. However, the concentrations in these sources are significantly lower than the therapeutic doses used in clinical trials, making it impractical to rely on dietary intake alone to achieve similar effects^[12]^. Supplementation of PEA is a feasible therapeutic approach to increase the systemic and tissue levels of PEA and restore the body's normal homeostasis, because endogenous PEA levels are generally reduced and insufficient to combat the chronic allostatic load observed in various chronic inflammatory disorders. Numerous nutraceuticals as well as health and dietary supplements marketed in various countries contain PEA as the main ingredient^[10]^.

Despite its long history of application in various conditions, very few studies are available that describe the pharmacokinetic profile of oral PEA supplementation. As PEA is highly lipophilic, few studies have described its solubility as "like trying to dissolve stones", making its oral absorption profile highly dependent on its molecular size^[12]^. Because of the lack of dedicated studies, the pharmacokinetic profile of PEA is highly theoretical and needs clinical validation. Aside from the oral route of administration, a study involving rabbits was conducted to evaluate the ocular pharmacokinetic profile of PEA after topical administration^[13]^. It was observed that topical ocular administration of PEA in a nano-lipid carrier system led to the detection of PEA in the rabbit retina within 180 minutes after administration, while the topical instillation of PEA in aqueous suspension led to only minor detectable quantities in the lens and vitreous humor, and none in the retina^[13]^. These observations indicate that PEA's absorption may be highly dependent on its molecular size during administration; consequently, various micronized and nano-sized PEA formulations have been developed to improve the PEA absorption profile^[12]^. Consistently, an experimental pharmacokinetic study showed a significantly better absorption profile of um-PEA than the conventional PEA formulation, confirming the influence of particle size on PEA's absorption^[14]^.

Following absorption, the next step involves the distribution profile of PEA in pharmacokinetics. Because of its lipophilic nature, PEA has a high volume of distribution and is well distributed in various regions of the body. Orally administered PEA may also reach the hypothalamus region of the brain^[12]^.

Apart from absorption and distribution, various studies have evaluated the pathways by which PEA is metabolized in the body. PEA is primarily metabolized through enzymatic pathways involving hydrolysis. Fatty acid amide hydrolase (FAAH) is the primary enzyme responsible for the hydrolysis of PEA, breaking it down into palmitic acid and ethanolamine. FAAH is widely distributed in tissues, particularly in the brain and liver. Another enzyme involved in the hydrolysis of PEA is N-acylethanolamine acid amidase, which is more selective for PEA and is primarily located in macrophages and other immune cells. It also converts PEA into palmitic acid and ethanolamine. Furthermore, palmitic acid may be incorporated into phospholipids or oxidized by beta-oxidation^[12]^.

While the end-products of PEA's metabolism are verified to be palmitic acid and ethanolamine, little has been established regarding the excretion profile of PEA based on the currently available literature. Although certain literature cites PEA's excretion *via* renal pathways, this needs to be verified through well-designed studies^[12]^.

### Nutrition and endocannabinoid system: Link in physiology and pathology

Balanced diet and nutrition are major determinants that affect the endocannabinoid system. Simultaneously, changes in the endocannabinoid system are thought to significantly influence appetite, eating behavior, and overall energy balance^[15]^. Studies have identified a link between dietary nutrition (mainly fatty acids) and endocannabinoid levels^[16]^. Anandamide (AEA) and 2-arachidonoylglycerol (2-AG), the most widely studied endocannabinoids, are derived from a polyunsaturated fatty acid (*i.e.*, arachidonic acid), suggesting that dietary fatty acids may affect the endocannabinoid synthesis pathway^[16–17]^. In a recent clinical study involving participants with habitual sedentary lifestyles, the consumption of a Mediterranean diet significantly altered endocannabinoid levels by positively altering the gut microbiome and increasing the level of *Akkermansia muciniphila*, a mucin-degrading probiotic^[18]^. In the same study, it was observed that participants with different baseline endocannabinoid levels experienced varying effects from the Mediterranean diet. Participants with low plasma oleoylethanolamide (OEA)/PEA levels had improved insulin resistance, while those with high plasma OEA/PEA levels showed a significant reduction in systemic inflammation^[18]^. Similarly, a clinical study involving binge alcohol consumers showed that acute alcohol exposure was associated with alterations in serum fatty acid derivatives, including endocannabinoid levels^[19]^. This evidence suggests that dietary nutrition may significantly modulate the endocannabinoid levels and endocannabinoid system activity by altering molecular signaling pathways and the gut microbiome.

Similarly, the endocannabinoid system regulates appetite, food intake, and energy metabolism by initiating complex signaling pathways and altering the levels of orexigenic and anorexigenic mediators, including leptin and ghrelin^[15–16,20]^. The presence of CB_1_ receptors on vagal nerve neurons suggests a potential role of endocannabinoids in the cholecystokinin signaling pathway, thereby modulating brain-derived appetite control^[16,20]^. Circulating endocannabinoids also have differential effects on adipose tissues and hepatocytes, ultimately modulating the levels of free fatty acids in the systemic circulation and the fate of energy metabolism in the body^[15–16]^.

As the endocannabinoid system plays an influential role in various pathological conditions, research has begun to identify endogenous and exogenous (natural and synthetic) compounds that may alter the activity of the endocannabinoid system to provide therapeutic benefits^[21]^. Various phytocannabinoids (including delta-9-tetrahydrocannabinol, cannabidiol, and related compounds), synthetic cannabinoids (including dronabinol and nabilone), and certain agents currently under evaluation for their safety and clinical efficacy have been developed to stimulate the endocannabinoid system and provide therapeutic effects in different pathological conditions^[21]^.

## PEA: Role in normal physiology and its mechanism in clinical applications

### Role of PEA in MetSyn and obesity

"Metabolic conditions" is an umbrella term used to describe various conditions in which alterations in one or more metabolic pathways ultimately impair normal metabolism^[22]^. Metabolic disorders negatively alter the body's normal signaling pathways as well as the processing and distribution of macronutrients, including fats, carbohydrates, and proteins. Obesity is a chronic metabolic disorder characterized by excessive growth of adipose tissue and the accumulation of fat in non-adipose tissues. It is usually accompanied by dyslipidemia, which increases cholesterol and fatty acid levels. Additionally, insulin resistance and hypertension are commonly observed in obese individuals^[23]^. Given the endocannabinoid system's key role in energy and metabolic homeostasis, abnormalities in this system play a central role in the initiation and progression of obesity and MetSyn, possibly by altering appetite and energy balance, increasing lipid production and fat accumulation, impairing glucose and insulin production (which leads to insulin resistance), and altering gut microbiome activity^[24–25]^. PEA may be a promising therapeutic option for the dietary management of MetSyn conditions^[26]^.

The supplementation of ultramicronized-PEA (um-PEA) increased the conversion of white adipose tissues to beige adipose tissues, along with enhanced thermogenic markers and leptin signaling in high-fat diet-induced obese mice, by activating peroxisome proliferator-activated receptor-alpha (PPAR-α)^[27]^. um-PEA altered energy homeostasis, prevented fat accumulation, and modulated adipocyte differentiation, suggesting its potential role in obesity^[27]^. A clinical study demonstrated altered endocannabinoid system activity in obesity and metabolic conditions, revealing significant differences in endocannabinoid levels between insulin-resistant obese postmenopausal women and their insulin-sensitive counterparts^[28]^. The study also indicated that high serum PEA levels might be associated with weight loss by promoting a favorable inflammatory environment^[28]^. Because gut microbiome dysfunction plays a crucial role in the progression of systemic inflammation and obesity^[25]^, um-PEA was found to improve the gut microbiome by enhancing PPAR-α activity and its association with tryptophan metabolism^[29]^.

PEA's role in metabolic health also extends to liver function, which is one of the most important metabolic organs in the body. The presence of endocannabinoid receptors on hepatic cells suggests that PEA administration may directly or indirectly (*via* the entourage effect) modulate hepatic metabolism. In a preliminary study, PEA therapy significantly altered the levels of fatty acids, glucose, amino acids, and hepatic growth rate, demonstrating the positive role of PEA in hepatic health^[30]^.

Fatty liver disease is a metabolic condition correlated with increased intrahepatic fatty acid accumulation, which activates a reactive chain within the liver that ultimately leads to increased oxidative stress and inflammation, damaging hepatic cells and resulting in non-alcoholic steatohepatitis (NASH). The sustained hepatic inflammation further activates hepatic stellate cells, thereby progressing NASH to hepatic fibrosis and further to hepatocellular carcinoma and end-stage liver disease^[31]^.

PEA administration was observed to reduce the activation of hepatic Kupffer and stellate cells in an *in vitro* study using a hepatic stellate cell line, while in a carbon tetrachloride-induced liver fibrosis animal model, PEA therapy significantly reduced hepatic type Ⅰ collagen deposition and overall hepatic fibrosis rate^[32]^. Similarly, PEA therapy was associated with a significant reduction in hepatic lipid accumulation and insulin resistance, while also increasing total energy expenditure^[33]^.

Although PPAR-α is the principal target for PEA's activity, some studies have highlighted PPAR-α-independent hepatoprotective actions as well. In an experimental NASH study, um-PEA supplementation upregulated the expression of PPAR-α and the phosphorylation level of adenosine monophosphate (AMP)-activated protein kinase (AMPK), while reducing the expression of acetyl-CoA carboxylase-1 (ACC1) and CD36 in hepatic cells^[34]^. Both ACC1 and CD36 play important roles in hepatic lipogenesis, fatty acid infiltration, and hepatic cancer development and progression^[35–36]^.

The reduction of ACC1 and CD36 expression by um-PEA correlated with reduced inflammatory mediators, decreased oxidative stress, and improved hepatic antioxidant potential. These changes, in turn, improved liver function, prevented lipid accumulation, and significantly prevented the pathological progression of hepatic fibrosis^[34]^. Overall, these positive results highlight the hepatoprotective activity of PEA in fatty liver and NASH conditions.

PEA also plays a crucial role in gastrointestinal health by interacting with the gut microbiome, which is essential for maintaining normal gastrointestinal function and the endocannabinoid environment^[37]^. In an *in vitro* study, the addition of fatty acid endocannabinoids to a gut microbiome-like culture significantly increased beneficial microbes while reducing the pathogenic microbes^[37]^. Because the fatty acid endocannabinoids share structural similarities with long-chain fatty acids, antimicrobial agents produced by gut microbes, it is postulated that the gut microbiome consumes the fatty acid endocannabinoids to form and release long-chain fatty acids. Additionally, as a part of the cellular membrane, fatty acid endocannabinoids may improve the membrane stability of the gut microbiome and the intestinal epithelial cells, thereby improving gut barrier integrity and overall gut health^[37]^. PEA has been reported to prevent intestinal damage and reduce the absorption of lactulose and mannitol^[38]^. These observations highlight the importance of endocannabinoid signaling in maintaining intestinal health, suggesting that exogenous PEA supplementation may improve overall gut health.

### Role of PEA in cardiovascular conditions

The endocannabinoid system is widely distributed in the cardiovascular system, with receptors present in the cardiac muscle, endothelial and smooth muscle cells, as well as on the pre-synaptic sympathetic nerve terminals that innervate the cardiovascular system^[39]^. The positive role of PEA (either as a single um-PEA ingredient or in combination with other ingredients like polydatin and baicalein) has been evaluated in various experimental vascular injury and myocardial ischemia animal models^[40–42]^. PEA supplementation has been correlated with significant reductions in vascular injury-induced inflammatory processes, reperfusion-associated myocardial injury, cellular apoptosis, and the expression and levels of immune adhesion molecules^[40–42]^. Similarly, in an experimental lipopolysaccharide-induced thrombosis animal model, um-PEA supplementation reduced the blood coagulation markers and prevented the deposition of fibrin by reducing the proinflammatory biomarkers^[43]^. Hypertension is a major cardiovascular disease that severely impacts individual health. As a potent PPAR-α agonist, PEA has been shown to improve blood pressure and prevent hypertension-related kidney damage in spontaneously hypertensive rats^[44]^. Additionally, PEA supplementation was correlated with an improvement in endothelium-derived hyperpolarizing factor activity, resulting in the vasodilation of arteries^[44]^. In the same study, it was observed that PEA supplementation significantly reduced the expression of angiotensin receptor 1 and angiotensin-converting enzyme, indicating that PEA may prevent hypertension by acting on the angiotensin-signaling pathway as well^[44]^. Furthermore, by interacting with the vascular CB_1_, transient receptor potential vanilloid-1, and probably G protein-coupled receptor 55 receptors, PEA modulates the sympathetic stimulation-related vascular contractile responses, which further supports the anti-hypertensive potential of PEA^[45]^.

### Role of PEA in the urinary system

The kidneys are major organs involved in the elimination of metabolic by-products and toxins, regulating body volume, and maintaining electrolyte balance and homeostasis^[46]^. With the increasing prevalence of acute kidney disease and chronic kidney disease (CKD), the search for new therapies to improve renal health has increased in recent decades. The cannabinoid receptors have demonstrated their role in maintaining normal renal hemodynamics and overall health, while alterations in the renal endocannabinoid system are associated with various renal complications^[46]^. In a renal ischemia/reperfusion (I/R)-induced renal injury animal model, PEA supplementation significantly attenuated renal dysfunction. Similarly, in an experimental study involving mice subjected to renal I/R injury, supplementation of PEA along with silymarin was found to significantly attenuate renal dysfunction and histological damage, compared with silymarin alone^[47]^. PEA potentially regulates the nuclear factor kappa-B (NF-κB) signaling pathway, reducing the expression of pro-inflammatory cytokines and improving the expression of antioxidants, thereby reducing oxidative stress and exerting a renoprotective effect^[47]^. Similarly, in a study involving hypertensive rats, PEA therapy reduced the expression of cytochrome P450 (CYP) hydroxylase CYP4A, epoxygenase CYP2C23, and soluble epoxide hydrolase in renal tissues, indicating reduced inflammation and renoprotective effects^[48]^. Additionally, PEA reduced the expression of key enzymes synthesizing reactive oxygen species and reactive nitrogen species, including renal nicotinamide adenine dinucleotide phosphate hydrogen (NADPH) oxidase and inducible nitric oxide synthase (iNOS), thus reducing oxidative stress and maintaining a normal renal homeostatic balance^[47–48]^.

Diabetic nephropathy is one of the most common diabetic renal complications, which involves chronic systemic low-grade inflammation and oxidative stress that may lead to CKD^[49–50]^. In an I/R-induced renal damage animal model, PEA therapy attenuated the renal dysfunction by activating PPAR-α, thereby reducing inflammation and renal damage^[51]^. Similarly, in hypertensive rats, PEA therapy improved the angiotensin-1/angiotensin-2 balance and the renin-angiotensin-aldosterone system activity, thereby improving blood volume and blood pressure as well as overall renal hemodynamics^[51]^. In a contrast-induced nephropathy animal model, um-PEA therapy significantly reduced renal inflammation, thereby attenuating glomerular dysfunction and renal parenchymal damage^[52]^. This evidence highlights the potential of PEA in preventing the progression of CKD by improving the overall inflammatory environment.

### Role of PEA in the nervous system and brain health

Stroke is a medical condition related to the blockage of blood supply to certain areas of the brain, which may be caused by a vascular block (ischemic stroke) or a sudden rupture of the cranial blood vessel, leading to hemorrhage and reduced cranial blood supply (hemorrhagic stroke)^[53]^. Ischemic stroke is related to a series of events that lead to the disruption of the blood-brain barrier (BBB) integrity and cerebral inflammation^[54]^. In a middle cerebral artery occlusion-induced stroke animal model, PEA therapy significantly prevented brain histological and cellular damage by reducing inflammatory cytokine levels^[55]^. Additionally, by activating PPAR-α, PEA therapy prevented the expression of phosphorylated Jun kinase and NF-κB, thereby inhibiting cellular apoptosis, ischemia-induced cerebral damage, and neurobehavioral alterations^[55]^. Similarly, in a cerebral ischemia and reperfusion-induced stroke animal model, PEA therapy was correlated with improved neurological function but decreased inflammatory cytokine expression levels, volume of infarction, and cerebral edema levels^[56]^. PEA was found to prevent early BBB disruption events by inhibiting the activation of Rho-associated protein kinase (ROCK) and myosin light chain (MLC) signaling, which is a BBB-protective activity independent of PPAR-α signaling^[56]^. Molecular studies have demonstrated that the ROCK/MLC signaling pathway disrupts BBB integrity by increasing the expression of pro-inflammatory cytokines and the formation of cellular adhesion complexes^[57]^. Hence, the PEA-mediated inhibition of ROCK/MLC signaling may offer a new therapeutic avenue to prevent BBB disruption during ischemic stroke^[56]^. Stroke is correlated with increased neuroinflammation, which further activates a cascade of inflammatory signaling pathways, causing additional damage to cerebral tissues. In an ischemia-induced cerebral injury model, PEA activated PPAR-α and reduced neuroinflammation by promoting macrophage polarization from the pro-inflammatory M1 phenotype to the anti-inflammatory M2 phenotype^[58]^. A similar neuroprotective effect was observed in a middle cerebral artery occlusion-induced brain injury model by um-PEA with luteolin (PEALut)^[59]^. To support the experimental findings, a clinical trial evaluated the effectiveness of PEALut in patients with acute ischemic stroke who were undergoing thrombolytic treatment^[60]^. Patients receiving PEA supplementation along with standard therapy showed significantly better recovery and cognitive improvement compared with those in the standard group, indicating that PEA is a safe and effective therapy that may reduce the severity of neuroinflammation and improve the effectiveness of standard therapies for the better management of acute ischemic stroke conditions^[60]^.

Migraine is a condition characterized by recurrent impulsive headaches of unknown origin, with various evidence suggesting that neuroinflammation plays an important role^[61]^. The trigeminovascular system is one of the major systems involved in migraine, where the sequential activation of the trigeminovascular system causes the release of various neurogenic peptides from trigeminal endings into the meningeal vessels, leading to the release of inflammatory mediators from the meninges^[62]^. Prolonged inflammation reduces the nociceptor activation threshold, leading to central and peripheral sensitization^[62]^. Some studies have found that the levels of AEA, a major endocannabinoid lipid, were reduced in the serum and cerebrospinal fluid of migraine patients, highlighting that alterations in the endocannabinoid system may contribute to migraine pathophysiology, and therapies targeting this system activity may be beneficial^[62]^. In a study involving children with recurrent migraine without aura, three months of um-PEA therapy significantly reduced the frequency and severity of migraine attacks, supporting the use of PEA as prophylactic therapy for migraines^[63]^. Similarly, in a study including subjects with migraine with aura, 90 days of um-PEA therapy combined with non-steroidal anti-inflammatory drugs (NSAIDs) provided greater pain relief compared with NSAIDs alone, further supporting the role of PEA for migraine prophylaxis and treatment^[64]^. In another open-label study involving patients with migraine with or without aura, the supplementation of a PEA-containing nutraceutical reduced the frequency and intensity of migraine attacks as well as the use of analgesic medications^[65]^. While PEA has been shown to increase the levels of AEA and show neuroprotective, anti-inflammatory, and analgesic activity, more studies are required to fully elucidate its mechanistic role in migraine conditions^[62,66]^.

Mild cognitive impairment (MCI) is a cognitive impairment disorder that does not significantly affect the normal daily life activities of an individual but is considered the pre-symptomatic stage of dementia. MCI is divided into two types: amnestic-MCI, in which only memory is affected, and non-amnestic-MCI, in which one or more cognitive domains other than memory are affected^[67]^. The potential of PEA in MCI has been demonstrated in a case study, in which an elderly female patient with amnestic-MCI showed a significant improvement in cognitive performance and cerebral perfusion (as evaluated using single-photon emission computed tomography) after nine months of PEALut therapy^[68]^. These findings highlight the neuroprotective efficacy of PEA, partly by resolution of neuroinflammation *via* PPAR-α agonism^[68]^.

Alzheimer's disease (AD) is a neurodegenerative condition that involves neuronal atrophy in the brain, leading to progressive memory loss and cognitive decline. The formation of amyloid beta (Aβ) plaques on the outer surface of the brain and hyperphosphorylated tau protein within neurons are the most common hallmarks of AD^[67]^. Various experimental studies have shown that PEA may counteract neuroinflammation and neuronal damage by activating PPAR-α, while protecting neurons by preventing Aβ-induced reactive gliosis^[67]^. In an Aβ-induced neuronal damage animal model, PEA significantly reduced neuronal degeneration and improved learning and memory functions^[67]^. By activating PPAR-α, PEA may attenuate the neuroinflammation and neuronal atrophy observed in AD. In an *in vitro* study, the addition of Aβ in an astrocyte cell line prevented the release of astrocyte-derived growth factors, reduced the oligodendrocyte maturation rate, and increased inflammatory cytokine release, while PEALut therapy counteracted the Aβ-induced effects, probably by PPAR-α activation^[69]^. These results strongly support further clinical evaluation of PEA supplements in AD patients.

Parkinson's disease (PD) is an age-related neurodegenerative disease associated with the loss of dopaminergic neurons in the nigrostriatal pathway, leading to the characteristic physical symptoms of excessive shaking, muscle stiffness, and walking and balance coordination impairment^[67]^. Various studies have suggested an important role of neuroinflammation in PD pathogenesis^[70]^. In a 1-methyl-4-phenyl-1,2,3,6-tetrahydropyridine-induced neurotoxicity PD animal model, PEA significantly prevented the loss of brain dopaminergic neurons and the development of motor deficits, while promoting neurogenesis, probably by PPAR-α activation and anti-inflammatory mechanisms^[67]^. Similarly, the motor disturbances caused by unilateral intra-striatal 6-hydroxydopamine injection were attenuated following PEA administration, which reduced neuroinflammation-induced neurotoxicity and neuronal death^[67]^. In clinical settings, supplementation with um-PEA and PEALut in PD patients significantly improved both motor and non-motor symptoms, reduced dyskinesia of the legs and trunk, and lowered the incidence of camptocormia, a PD-associated spinal deformity^[71–72]^. These findings underscore the potential of PEA as a supportive therapy for PD management.

Huntington's disease (HD) is a rare autosomal dominant disorder caused by a mutation in the *HTT* gene on chromosome 4, which leads to an overproduction of the mutant huntingtin (m-Htt) protein that aggregates in brain regions, causing neuronal dysfunction and degeneration^[73]^. HD is characterized by the loss of GABAergic medium spiny neurons, astrogliosis, motor symptoms (primarily chorea), dementia, psychiatric disturbances, and premature mortality^[67,74]^. Neuroinflammation plays an important role in the HD disease progression and symptom severity^[75]^. In a transgenic mouse model of HD, striatal levels of major endocannabinoids, including PEA, were significantly reduced, suggesting that the endocannabinoid system dysregulation contributes to HD pathogenesis and neuroinflammation^[67]^.

Neuropathy is a functional somatic syndrome characterized by chronic pain without obvious structural damage. Tissue injury, infections, disease, and stress may cause peripheral nerve damage, initiating inflammation that activates nociceptors and causes persistent pain^[76]^. Chronic inflammation reduces the nociceptor activation threshold and increases pain perception, a phenomenon known as pain sensitization^[76]^. PEA is considered a disease-modifying agent in neuropathy because of its anti-nociceptive and anti-inflammatory properties^[77]^. In chronic constriction injury-induced neuropathy, PEA supplementation significantly reduced pain and improved neuronal health by PPAR-α activation and modulation of inflammatory pathways^[77]^. Data from clinical studies also showed that PEA, along with other standard therapies, such as gabapentinoids, opioid analgesics, transdermal fentanyl and buprenorphine, and paracetamol, resulted in clinically meaningful reductions in pain in patients with various chronic painful conditions, including radiculopathy^[78]^. By activating PPAR-α, PEA suppressed the nociceptive signaling, demonstrating its efficacy in reducing pain and improving the quality of life in more than 6000 patients suffering from various painful conditions^[78]^.

Amyotrophic lateral sclerosis (ALS) is a CNS neurodegenerative condition primarily caused by mutations in the *TARDBP* gene, leading to the destruction of TAR DNA-binding protein-43 (TDP-43) and aggregation in motor neurons, which presents as a motor neuron dysfunction and leads to progressive weakness of voluntary muscles involved in limb movement, difficulty in swallowing, speaking, and respiratory function, along with cognitive changes like poor working memory, lack of interest, irritability, abnormal eating behavior, and altered language fluency^[79]^. The neurodegeneration in ALS is related to significant spinal inflammation driven mainly by mast cells and microglia^[67]^. Two clinical studies have demonstrated improvement in muscle tone, motor skills, and respiratory function in ALS patients receiving PEA therapy, likely due to reduced mast cell and microglial activity^[67]^.

Multiple sclerosis (MS) is an autoimmune demyelinating disease characterized by progressive immune-mediated attacks on the neuronal myelin sheath and inflammation, which further triggers the recruitment of more immune cells in the brain, causing further damage to the myelin sheath^[80]^. The anti-inflammatory properties of PEA have been evaluated in experimental autoimmune encephalomyelitis, an animal model of MS, in which intraperitoneal PEA administration reduced inflammation, demyelination, neuronal degeneration, and behavioral impairment^[67]^. In a clinical study, um-PEA therapy significantly reduced serum cytokine levels and improved overall quality of life in MS patients, suggesting its therapeutic potential for MS management^[81]^.

### Role of PEA in the muscular system

Fibromyalgia (FM) is a condition related to widespread pain and tenderness in deep tissues and muscles, fatigue, and reduced muscle strength^[82]^. Numerous studies have suggested that chronic inflammation plays an important role in increasing nociceptive sensations and reducing pain thresholds in FM^[83]^. PEA modulates inflammation by activating PPAR-α and reducing the activity of non-neuronal cells (mast and glial cells), thereby reducing painful sensations. In a clinical study involving FM patients previously treated with duloxetine + pregabalin (DuoPre), the um-PEA add-on therapy showed a greater reduction in pain and FM tender points, compared with the DuoPre alone therapy^[84]^. In another similar clinical study, FM patients previously under DuoPre therapy were randomized to receive a combination of PEA and acetyl-L-carnitine alongside DuoPre, or to continue DuoPre therapy only. The addition of PEA significantly enhanced pain reduction and improved quality of life, compared with the DuoPre therapy alone, supporting the hypothesis that PEA, by reducing inflammation, provides an analgesic effect and may also improve the efficacy of standard therapeutic regimens^[85]^. Another clinical study demonstrated significant pain reduction and improved overall quality of life in FM patients with um-PEA therapy^[86]^.

### Role of PEA in ophthalmic health

The basic pharmacology and functioning of the retina show high similarity to CNS neuronal cells, starting from receiving signals to converting them into electrical impulses^[87]^. Hence, as the endocannabinoid system has a crucial role in maintaining CNS health, it is plausible that it also contributes significantly to retinal and overall ocular health^[87]^. Degenerative retinal disease is a condition related to progressive retinal damage, leading to retinopathy and loss of vision. Among the various risk factors, chronic inflammation in the retinal area plays a central role in nearly all types of retinopathies, including diabetic retinopathy, glaucoma, and age-related macular degeneration (AMD)^[88]^.

Glaucoma is a condition in which elevated intraocular pressure (IOP) progressively damages the optic nerve, potentially leading to blindness^[89]^. Among the other determinants, certain evidence supports the role of retinal inflammation in increasing IOP in glaucoma^[89]^. The possible retinoprotective effects of PEA have been explored in various experimental studies^[89]^. In a study involving the assessment of human eye tissues obtained from normal and glaucomatous patient donors, PEA levels were significantly decreased in the ciliary body of glaucomatous patients, suggesting that PEA may help maintain normal IOP by regulating ciliary body function^[90]^. To support this hypothesis, various clinical studies showed that PEA therapy effectively reduced IOP and improved related visual field activity, tear properties, endothelial functioning, and retinal functionality^[91–94]^.

Diabetic retinopathy is the major ophthalmic complication of diabetes, affecting around 30%–40% of diabetics^[95]^. As PEA has anti-inflammatory, retinoprotective, and antioxidant potential, its therapy in streptozotocin-induced diabetic rats significantly prevented the retinal damage and preserved the blood-retinal barrier by reducing inflammation levels^[96]^. Future clinical trials are warranted to confirm these findings and evaluate the therapeutic potential of PEA in human diabetic retinopathy.

Age-related macular degeneration is characterized by progressive and irreversible damage to the retina and associated tissues^[97]^. AMD is characterized into two types: non-neovascular AMD ('dry' type) and neovascular AMD ('wet' type)^[97]^. Inflammation is thought to be one of the main factors in AMD pathogenesis, as it contributes to choroidal neovascularization and ocular tissue atrophy^[98]^. In both an oxygen-induced retinopathy model and a very low-density lipoprotein receptor knockout (*Vldlr*^*−/−*^) mouse model of retinopathy, PEA therapy significantly reduced profibrotic retinal changes. By activating PPAR-α, it reduced inflammation and suppressed the development of Müller gliosis, providing preliminary evidence for the beneficial role of PEA in preventing retinal damage observed in AMD^[99]^. These studies suggest the potential therapeutic role of PEA in various ophthalmic conditions, warranting further investigation in clinical settings.

### Role of PEA in the osseous system

Bone is a dynamic organ that is constantly undergoing formation and breakdown to maintain the homeostasis of the bone and the body. One study has identified the distinct role of the endocannabinoid system in regulating bone health^[100]^. A supplement containing dispersible PEA has shown a significant reduction in both morning and evening joint pain^[101]^. Similarly, in osteoarthritis, PEA therapy significantly reduced levels of pro-inflammatory mediators, including leukotriene B4, tumor necrosis factor-α, interleukin-1β, and prostaglandin E2, as well as cartilage-degrading enzymes such as matrix metalloproteinase-2 (MMP-2), MMP-3, MMP-9, and MMP-13. These effects contributed to reduced joint swelling and cartilage degradation^[102]^. These findings highlight the anti-inflammatory and chondroprotective potential of PEA in promoting bone and joint health.

### Role of PEA in oncological conditions

First described in the 1970s, the anti-neoplastic properties of cannabinoids were observed when delta-9-tetrahydrocannabinol, the active compound in *Cannabis sativa*, was shown to reduce leukemia cell growth^[103]^. Since then, the anti-neoplastic potential of endogenous and phyto-cannabinoids, including PEA, has been extensively studied.

Breast cancer is the most common type of cancer in female population. Although the research on PEA in breast cancer is still in its infancy, an *in vitro* study using human breast cancer cells showed that PEA inhibited the activity of fatty acid amide hydrolase, the enzyme responsible for the degradation of AEA. This inhibition potentiated the anti-neoplastic effect of AEA by increasing the activation of CB receptors. Additionally, PEA reduced the expression and signaling of the nerve growth factor/TrkA pathway, which typically activates the Ras/MAPK cascade, a key driver of cancer cell proliferation, invasion, and metastasis^[104–105]^.

Colorectal cancer is one of the most common cancers globally, and among its various determinants, alterations in energy homeostasis have been associated with the development and/or progression of colorectal cancer^[106]^. PEA, as a modulator of overall energy homeostasis, has been shown to upregulate the cyclin B1/cyclin-dependent kinase-1 pathway, resulting in cell cycle arrest and DNA fragmentation in a pre-clinical murine model of colon cancer, potentially through the activation of PPAR-α and GPR-55^[107]^. This effect was correlated with a significant reduction in preneoplastic lesions and tumor formation in PEA-supplemented animals, which provides novel insights into the role of PPAR-α and GPR-55 in the cell cycle and the anti-neoplastic potential of PEA^[107]^.

Cervical cancer, predominantly caused by persistent human papillomavirus infection, is the third leading malignancy in the female population^[108]^. Among the various molecular mechanisms implicated in its pathogenesis, the ubiquitin-proteasome pathway (UPP)-mediated degradation of cell cycle regulatory proteins plays a key role^[109]^. UPP is a protein degradation pathway that involves the ubiquitin protein-mediated degradation of target proteins by the 20S-proteasome complex^[110]^. While the UPP system plays an essential role in the overall protein turnover rate, its uncontrolled activation and the destruction of cell-cycle regulatory proteins (namely cyclins, cyclin-dependent kinases, and cyclin-dependent kinase inhibitors) may lead to uncontrolled cellular proliferation and ultimately cancer^[110]^. In a preliminary *in vitro* study using human cervical cancer (HeLa) cells, exogenous PEA administration significantly inhibited the activity of the proteasomes and increased the caspase-3 activity, highlighting its potential anti-neoplastic effects^[111]^. These promising findings require further validation in pre-clinical animal models and well-designed clinical trials.

### Role of PEA in the immune system

The immune system is an important defense mechanism of the body. PEA first appeared on the market in the 1960s for the prophylactic treatment of influenza and the common cold^[10]^. By activating PPAR-α, PEA modulates immune responses by regulating macrophage activity and influencing the degranulatory process of mast cells. Additionally, through the "entourage effect", PEA may modulate the non-specific innate immune responses against bacterial and viral pathogens^[10]^. These immunomodulatory properties underpin the broad-spectrum anti-viral and anti-bacterial potential of PEA^[10]^. In patients with severe acute respiratory syndrome coronavirus-2 (SARS-CoV-2), PEA therapy significantly improved the olfactory response, probably through its anti-inflammatory activity^[112]^.

## Safety of PEA

**Figure 3:**
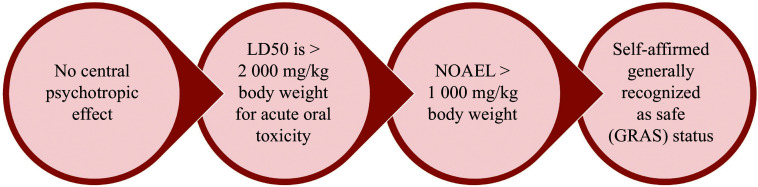
Safety of palmitoylethanolamide. Abbreviations: LD50, median lethal dose; NOAEL, no observed adverse effect level.

PEA has been shown to be both safe and effective in alleviating pain and improving the quality of life in over 6000 patients with chronic pain conditions. Clinical studies have consistently demonstrated significant pain reductions, along with improvements in overall well-being and daily functioning. This growing body of evidence supports the use of PEA as a reliable option for managing chronic pain and improving the quality of life in affected individuals^[78]^. The safety of PEA is further illustrated in ***Fig. 3***^[113–114]^.

## Conclusions and perspectives

Despite advancements in modern medicinal science, the role of balanced nutrition remains pivotal in maintaining a healthy, disease-free life. However, the global rise in nutrition imbalance is impressing at an alarming rate and is responsible for various alterations in normal physiological processes, leading to chronic disease conditions. One of the physiological pathways is the endocannabinoid system, which plays an important role in various disease conditions. With the advancement of molecular and nutritional research, it is now well-established that dietary nutrition and the endocannabinoid system have a significant bi-directional interaction. Based on this inference, the endocannabinoid system may have a great therapeutic potential in various disease conditions. This particular hypothesis has led to the development of drug candidates that may positively modulate the activity of the endocannabinoid system. The present review article has particularly highlighted the potential of palmitoylethanolamide, a "non-typical" endogenous cannabinoid, produced in the body when required in response to various stimuli. The in-depth literature review presented in this article provides substantial evidence for the potential role of palmitoylethanolamide in normal healthy conditions and its putative role in various disease conditions. However, despite large experimental and clinical investigations undertaken with positive results, many more domains still require exploration to fully support the therapeutic potential of palmitoylethanolamide. The present review article has also underscored some major research gaps that may guide future investigators in exploring the varied roles of palmitoylethanolamide and in designing experimental and clinical studies that might bridge these knowledge gaps.
